# Oral health-related quality of life in young adults born with unilateral cleft lip and palate after interdisciplinary treatment: a cross-sectional study with controls

**DOI:** 10.1093/ejo/cjaf076

**Published:** 2025-10-01

**Authors:** Mathias Lemberger, Marie Pegelow, Petra Peterson, Pernilla Larsson, Agneta Karsten

**Affiliations:** Division of Orthodontics, Department of Dental Medicine, Karolinska Institutet, Box 4064, Huddinge SE-141 04, Sweden; Department of Orthodontics, Public Dental Services Stockholm, Eastman Institute, Box 6031, Stockholm SE-102 31, Sweden; Division of Orthodontics, Department of Dental Medicine, Karolinska Institutet, Box 4064, Huddinge SE-141 04, Sweden; Department of Molecular Medicine and Surgery, Karolinska Institutet, Stockholm 171 77, Sweden; Department of Plastic and Craniofacial Surgery, Karolinska University Hospital, Stockholm SE-171 76, Sweden; Department of Prosthetic Dentistry, Odont Faculty, Malmo University, Malmö 214 21, Sweden; Division of Orthodontics, Department of Dental Medicine, Karolinska Institutet, Box 4064, Huddinge SE-141 04, Sweden

## Abstract

**Background:**

Outcome studies conducted on patients treated for cleft lip and palate (CLP) typically focus on clinical measures, while significantly fewer investigate patients’ subjective perceptions of the long-term outcomes.

**Objective:**

To evaluate oral health related quality of life (OHRQoL) as reported by young adults born with unilateral cleft lip and palate (UCLP).

**Material:**

The study sample consisted of 32 consecutive patients (mean age 19.1 years) born with UCLP that had undergone interdisciplinary treatment. This group was compared with two noncleft control groups: one orthodontically treated group (Ortho) (*n* = 32, mean age 18.7 years) and one nonorthodontically treated group (Control) (*n* = 31, mean age 18.6 years).

**Method:**

In this cross-sectional, questionnaire-based study, three instruments, the oral health impact profile-14 (OHIP-14), the jaw functional limitation scale-20 (JFLS-20), and the orofacial esthetic scale (OES) were administered during the final routine follow-up visit at 19 years of age.

**Results:**

No significant differences were found in JFLS-20 and OHIP-14 mean summary scores between the UCLP group and the noncleft groups. For OES, the UCLP group differed significantly from the Ortho group (*P* = 0.042) but not from the Control group.

**Conclusion:**

Young adults born with UCLP treated in an interdisciplinary team reported overall OHRQoL comparable to peers without a cleft and no history of orthodontic treatment. Their self-perceived orofacial appearance was similar to that of noncleft, nonorthodontically treated peers, but rated less favorable compared to noncleft peers who had received orthodontic treatment.

## Introduction

Many children born with unilateral cleft lip and palate (UCLP) undergo a comprehensive care program aimed at achieving good orofacial function and esthetics [1]. The program starts at birth and continues into adulthood, and is often delivered by dedicated interdisciplinary teams [2]. The team in Stockholm involves plastic surgeons, orthodontists, speech-language pathologists, psychologists, and other specialists who collaborate to coordinate care across developmental stages. In many highly developed countries, including Sweden, treatments are financed by the welfare system, while in other countries, families are responsible for identifying care providers and covering the costs themselves. Despite proper implementation of treatment programs, some residual conditions associated with being born with a cleft can remain. These may include nasal asymmetry and visible lip scars [3]. Growth disturbances of the maxilla and velopharyngeal function can also occur, leading to a concave facial profile and impaired speech [4, 5]. Orofacial function and appearance may influence how individuals perceive themselves and how they interact socially. While previous studies evaluating interdisciplinary treatment outcomes for children with cleft lip and palate (CLP) have primarily focused on objective clinical measures, significantly fewer studies have investigated patient satisfaction and self-perceived outcomes. Questionnaires are valuable tools for exploring patients’ subjective perceptions of treatment outcomes. Questionnaire-based studies have shown that having an orthodontic diagnosis negatively impacts OHRQoL, while orthodontic treatment tends to improve it [6, 7]. Studies using self-reported measures have indicated that individuals treated for UCLP are generally less satisfied with their lip and facial appearance than noncleft controls [8]. Among those treated for CLP, appearance has been shown to play a key role in overall quality of life [9], and many individuals express a desire to change aspects of their facial appearance [8]. Several studies have reported that OHRQoL is lower in individuals with repaired CLP [10] or clefts in general, when compared with population norms [11]. Females born with CLP appear to be more negatively affected than males [12, 13]. Silva et al. (2018) found that in nearly half of the participants, the cleft negatively impacted daily activities, particularly speech, smiling, and showing teeth without embarrassment. Lecommandeur et al. reported that psychological well-being in adolescents with UCLP was generally comparable to that of matched peers, although some reported lower psychological quality of life and less satisfaction with social support [14]. To our knowledge, no previous study has compared self-reported OHRQoL in young adults born with UCLP to two noncleft age-matched groups stratified by orthodontic treatment status. Therefore, the aim of the present study was to assess self-reported OHRQoL in young adults born with UCLP after receiving interdisciplinary care, in comparison with noncleft peers who had or had not received orthodontic treatment.

## Material and methods

In this cross-sectional questionnaire-based study, a consecutive cohort of patients born with UCLP were asked to participate at their final routine follow-up examination at 19 years of age. To be included, patients had to be born with UCLP without any other associated syndrome and treated according to the Stockholm cleft treatment protocol, during the time period when the children underwent surgery. The protocol outlines the standard management of patients with UCLP in Stockholm, specifying the timing and surgical techniques used at each stage of treatment. The initial surgery, the lip repair, was performed at 4 to 6 months of age using the Tennison–Randell technique [15], combined with nasal repair according to McComb [16 ]. Palatal repair was performed using the “minimal incision technique” at 12–14 months [17, 18]. Alveolar bone grafting was performed between ages 8 and 12, depending on tooth development and eruption of the adjacent tooth (lateral incisor or canine), using the Boyne and Sands technique [19] with autologous bone from the iliac crest. If a lateral incisor was present, the aim was to preserve it. If necessary, pregrafting orthodontic treatment was initiated in the mixed dentition. All patients received orthodontic treatment after the alveolar bone grafting procedure. The study cohort included patients born with UCLP (*n* = 32, 11 females, mean age 19.1 years, range 18.5–20), treated within the Stockholm interdisciplinary treatment team. This group was compared with two noncleft groups: The Ortho group (*n* = 32, mean age 18.7 years, range 18–19), was recruited from the Department of Dental Medicine, Division of Orthodontics and Pediatric Dentistry at the Karolinska Institutet, Huddinge, Stockholm, during a follow-up visit after completed orthodontic treatment. The Control group (*n* = 31, mean age 18.6 years, range 17–22) was recruited from the same department during their annual dental health examination. Inclusion criteria for the Ortho group were adolescents in the permanent dentition who had undergone orthodontic treatment and were scheduled for the routine dental examination at 19 years of age. For the Control group, inclusion criteria were adolescents in the permanent dentition with no history of orthodontic treatment and with a treatment need score below the threshold for publicly funded orthodontics, according to the treatment need index used in Stockholm.

The Regional Ethical Review Board in Stockholm approved the present study (Dnr [daybook no.] 2008/502-31-2, 2016/1663-32, 2017/874-31). After oral and written information about the study, the included patients signed a consent form and filled out the questionnaires with a pen on a paper.

Three validated questionnaires were used. Oral health impact profile-14 (OHIP-14) [20], jaw functional limitation scale-20 (JFLS-20) [21], and orofacial esthetic scale (OES) [22, 23].

OHIP-14 consists of 14 items across four domains, oral function (five items), orofacial pain (one item), orofacial appearance (one item), and psychosocial impact (seven items). Responses are recorded on a 5-point scale (never = 0, hardly ever = 1, occasionally = 2, fairly often = 3, very often = 4 or not applicable). The maximum total score is 56, where higher scores indicate worse OHRQoL.

JFLS-20 includes 20 items in three domains: mastication, vertical jaw mobility, verbal and emotional expressions. Each item uses an 11-point scale (0 = no limitations to 10 = extreme limitation) with a maximum total score of 200, indicating extreme limitations.

The OES has eight items assessing self-perceived orofacial esthetics on an 11-point scale (0 = very dissatisfied to 10 = very satisfied). The total maximum score for items 1–7 is 70, indicating very high satisfaction. Item 8 is a global question regarding overall esthetic perception [23, 24].

### Statistical analyses

A sample size estimation was performed based on the results from a previous study by Larsson et al. [20], with a 5% alpha error (α = 0.05) and a power of 80%, indicating that a sample size of 29 per group was needed. Descriptive statistics were calculated, including mean scores with standard deviations for each domain and mean summary scores with 95% confidence intervals for each questionnaire. Differences between the three groups were examined using one-way ANOVA followed by Tukey’s test to assess differences between the UCLP and Ortho groups, and between the UCLP and Control groups. All statistical analyses were performed using the Statistical package for Social Sciences (SPSS, ver 22; Chicago, IL, USA).

## Results

**Figure 1.**
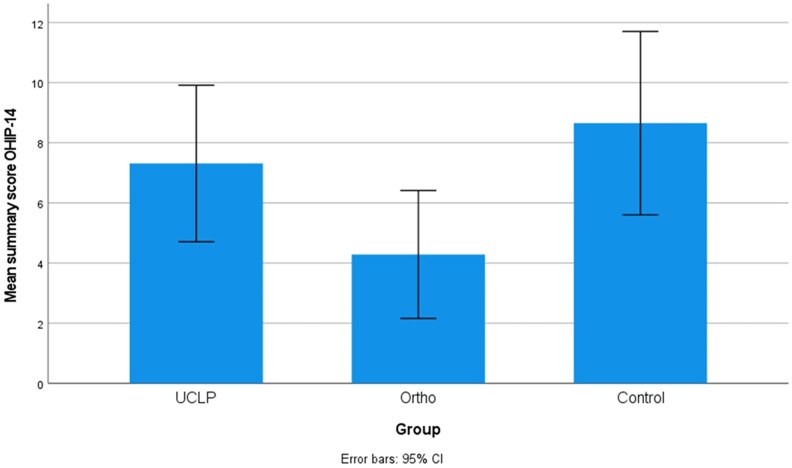
The oral health impact profile (OHIP-14) mean summary score with 95% confidence interval in the three groups: unilateral cleft lip and palate (UCLP), orthodontically treated group (Ortho), and nonorthodontically treated (Control).

**Figure 2.**
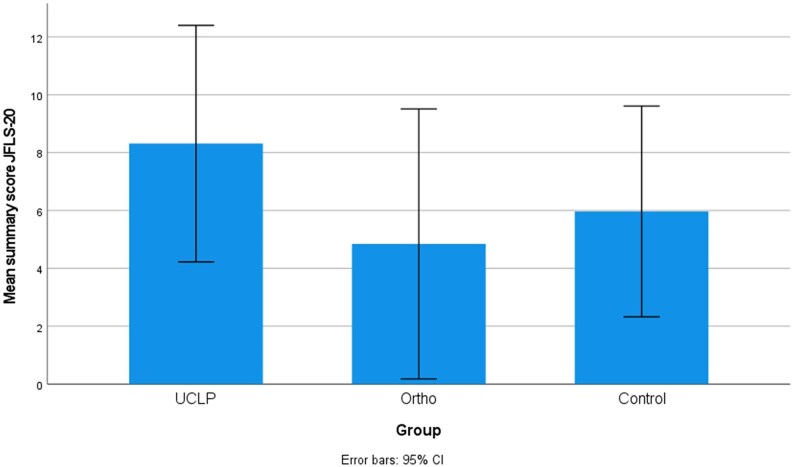
The jaw functional limitation scale 20 (JFLS-20) mean summary score with 95% confidence interval in the three groups: unilateral cleft lip and palate (UCLP), orthodontically treated group (Ortho), and nonorthodontically treated (Control).

**Figure 3.**
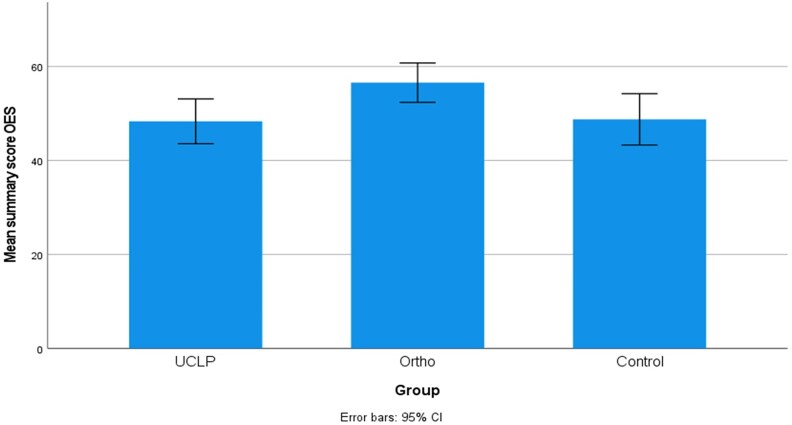
The orofacial esthetic scale (OES) mean summary score with 95% confidence interval in the three groups: unilateral cleft lip and palate (UCLP), orthodontically treated group (Ortho), and nonorthodontically treated (Control).

Mean summary scores with 95% confidence intervals for the three groups, UCLP, orthodontically treated group (Ortho), and nonorthodontically treated group (Control) are presented in Figs 1–3. Summary and subdomain scores for the UCLP and the two noncleft groups, both overall and divided by gender, are shown in Tables 1–3. One participant in the Control group stated that they did not identify as either female or male. Their responses were included in the overall analysis but excluded from the gender-specific comparisons.

**Table 1.** Summary and subdimensions mean scores from oral health impact profile (OHIP-14) in the three groups: unilateral cleft lip and palate (UCLP), orthodontically treated group (Ortho), and nonorthodontically treated (Control).

OHIP-14		UCLP		Ortho		Control		Significances	
	*n*	(x ± SD)	*n*	(x ± SD)	*n*	(x ± SD)	Overall	UCLP-ortho	UCLP-control
**OHIP-14 sum**	32	7.3 ± 7.2	28	4.3 ± 5.5	29	8.7 ± 8.0	0.061	0.224	0.736
**Male**	21	8.3 ± 8.3	16	3.8 ± 4.2	20	9.5 ± 9.0	0.079	0.186	0.877
**Female**	11	5.4 ± 4.2	12	4.9 ± 7.1	8	7.4 ± 5.5	0.629	0.981	0.734
**Oral function (F)**	29	2.6 ± 2.2	29	1.4 ± 1.6	29	2.0 ± 2.3	0.105	0.086	0.532
**Male**	18	3.2 ± 2.5	17	1.4 ± 1.5	20	2.4 ± 2.5	0.078	0.062	0.419
**Female**	11	1.6 ± 1.4	12	1.4 ± 1.9	8	1.4 ± 1.5	0.928	0.945	0.937
**Orofacial appearance (A)**	32	1. ± 1.1	31	0.3 ± 0.8	30	0.7 ± 0.8	0.020*	0.015*	0.564
**Male**	21	1.1 ± 1.2	17	0.1 ± 0.2	21	0.9 ± 0.9	0.002**	0.002**	0.58
**Female**	11	0.6 ± 0.7	14	0.6 ± 1.0	8	0.5 ± 0.5	0.914	1.000	0.931
**Orofacial pain (P)**	30	0.6 ± 0.9	31	0.6 ± 0.8	30	0.7 ± 0.8	0.968	0.995	0.987
**Male**	19	0.7 ± 1.0	18	0.8 ± 0.8	21	0.8 ± 0.9	0.906	0.949	0.903
**Female**	11	0.6 ± 0.7	13	0.4 ± 0.7	8	0.4 ± 0.5	0.786	0.811	0.833
**Psychosocial impact (PI)**	32	3.1 ± 4.0	28	2.0 ± 3.2	28	5.1 ± 4.8	0.020*	0.554	0.151
**Male**	21	3.4 ± 4.6	16	1.6 ± 2.4	19	5.2 ± 5.5	0.060	0.436	0.398
**Female**	11	2.6 ± 2.8	12	2.6 ± 4.1	8	5.1 ± 3.5	0.224	1.000	0.270

*p < 0.05.

**p < 0.01.

**Table 2.** Summary and subdimensions mean scores from jaw functional limitation scale 20 (JFLS-20) in the three groups: unilateral cleft lip and palate (UCLP), orthodontically treated group (Ortho), and nonorthodontically treated (Control).

JFLS		UCLP		Ortho		Control		Significances	
	*n*	(x ± SD)	*n*	(x ± SD)	*n*	(x ± SD)	Overall	UCLP-Ortho	UCLP-Control
**JFLS sum**	32	8.3 ± 11.3	32	4.8 ± 12.9	31	6.0 ± 9.9	0.471	0.452	0.698
**Male**	21	6.8 ± 12.0	18	6.6 ± 17.0	21	7.2 ± 11.1	0.987	0.998	0.994
**Female**	11	11.2 ± 9.9	14	2.6 ± 3.6	9	3.7 ± 6.8	0.012*	0.013*	0.059
**Mastication**	32	2.4 ± 3.7	32	1.3 ± 2.4	31	2.1 ± 3.6	0.369	0.358	0.926
**Male**	21	1.8 ± 3.8	18	1.6 ± 2.8	21	2.3 ± 3.8	0.802	0.983	0.882
**Female**	11	3.6 ± 3.2	14	0.9 ± 1.7	9	1.9 ± 3.5	0.065	0.053	0.349
**Vertical jaw mobility**	32	2.9 ± 4.9	32	0.8 ± 2.0	31	1.5 ± 3.7	0.084	0.074	0.305
**Male**	21	1.9 ± 4.9	18	1.1 ± 2.5	21	2.0 ± 4.3	0.767	0.82	0.997
**Female**	11	4.8 ± 4.6	14	0.5 ± 1.0	9	0.6 ± 1.1	0.001**	0.001**	0.005**
**Emotional**	28	3.1 ± 5.3	32	2.7 ± 10.1	31	2.3 ± 4.7	0.924	0.977	0.916
**Male**	18	3.3 ± 5.6	18	3.8 ± 13.4	21	2.9 ± 5.4	0.947	0.984	0.987
**Female**	10	2.6 ± 4.9	14	1.2 ± 2.6	9	1.2 ± 2.4	0.568	0.592	0.655

*p < 0.05.

**p < 0.01.

**Table 3.** Summary and subdimensions mean scores from Orofacial Esthetic Scale (OES) in the three groups: unilateral cleft lip and palate (UCLP), orthodontically treated group (Ortho), and nonorthodontically treated (Control).

OES		UCLP		Ortho		Control		Significances	
	*n*	(x ± SD)	*n*	(x ± SD)	*n*	(x ± SD)	Overall	UCLP-Ortho	UCLP-Control
**Overall OES**	32	6.94 ± 1.87	28	8.11 ± 1.60	31	7.32 ± 2.39	0.076	0.065	0.723
**Male**	21	6.81 ± 1.91	15	8.07 ± 1.75	21	7.38 ± 1.99	0.157	0.133	0.596
**Female**	11	7.18 ± 1.83	13	8.15 ± 1.46	9	6.89 ± 3.22	0.36	0.528	0.952
**Orofacial esthetic scale sum**	32	48.34 ± 13.24	28	56.57 ± 10.81	29	48.76 ± 14.38	0.028*	0.042*	0.991
**Male**	21	48.43 ± 12.34	15	56.27 ± 11.62	19	49.79 ± 10.89	0.127	0.125	0.928
**Female**	11	48.18 ± 15.46	13	56.92 ± 10.25	9	44.78 ± 20.09	0.162	0.349	0.872
**Face**	32	7.06 ± 1.93	28	8.71 ± 1.63	29	7.28 ± 2.81	0.010*	0.012*	0.923
**Male**	21	6.67 ± 1.98	15	8.67 ± 1.91	19	7.42 ± 2.85	0.045*	0.035*	0.56
**Female**	11	7.82 ± 1.66	13	8.77 ± 1.30	9	6.67 ± 2.83	0.057	0.461	0.392
**Profile**	32	6.3 ± 2.4	28	8.4 ± 2.0	29	7.2 ± 2.7	0.004**	0.003**	0.335
**Male**	21	6.1 ± 2.4	15	8.5 ± 2.1	19	7.6 ± 2.4	0.009**	0.009**	0.101
**Female**	11	6.8 ± 2.2	13	8.3 ± 2.1	9	6.0 ± 3.0	0.089	0.302	0.733
**Mouth**	32	6.4 ± 2.5	28	7.9 ± 2.2	29	7.5 ± 2.5	0.041*	0.040*	0.181
**Male**	21	6.3 ± 2.5	15	7.5 ± 2.4	19	8.0 ± 1.9	0.068	0.273	0.063
**Female**	11	6.5 ± 2.7	13	8.4 ± 2.0	9	6.1 ± 3.2	0.091	0.18	0.953
**Tooth alignment**	32	6.0 ± 2.5	28	8.1 ± 2.4	30	6.6 ± 2.7	0.061	0.172	0.858
**Male**	21	7.3 ± 2.3	15	8.2 ± 2.4	20	6.7 ± 2.3	0.185	0.525	0.667
**Female**	11	6.3 ± 2.7	13	8.1 ± 2.4	9	6.1 ± 3.4	0.18	0.265	0.991
**Tooth shape**	32	7.6 ± 2.2	28	8.5 ± 2.1	30	6.8 ± 2.7	0.025*	0.32	0.35
**Male**	21	7.6 ± 2.1	15	8.7 ± 1.4	20	6.9 ± 2.4	0.042*	0.294	0.458
**Female**	11	7.6 ± 2.3	13	8.3 ± 2.7	9	6.3 ± 3.4	0.282	0.83	0.564
**Tooth color**	32	6.9 ± 2.2	29	6.6 ± 2.4	30	5.6 ± 2.5	0.09	0.883	0.089
**Male**	21	7.1 ± 1.8	16	6.7 ± 2.6	20	5.6 ± 2.3	0.088	0.849	0.081
**Female**	11	6.5 ± 2.8	13	6.5 ± 2.1	9	5.9 ± 3.0	0.818	1.000	0.844
**Gingiva**	32	7.1 ± 2.5	28	8.0 ± 1.8	30	7.9 ± 2.2	0.208	0.229	0.355
**Male**	21	7.3 ± 2.0	15	7.6 ± 2.1	20	7.9 ± 2.2	0.734	0.925	0.711
**Female**	11	6.6 ± 3.4	13	8.5 ± 1.1	9	7.7 ± 2.4	0.173	0.148	0.612

*p < 0.05.

**p < 0.01.

### Oral health impact profile 14

There were no significant differences in mean summary OHIP-14 scores between the UCLP group and the two noncleft groups, Table 1. Two subjects in the UCLP group, two in the Ortho group and seven in the Control group reported a mean summary score of ≥15. A significant difference was found among males in the orofacial appearance subdomain, with higher scores in the UCLP group compared to the Ortho group (*P* = 0.002), Table 1.

### Jaw functional limitation scale

There were no significant differences in the mean summary score of JFL-20 between the UCLP group and the two noncleft groups, Table 2. Five subjects in the UCLP group, one in the Ortho group and four in the Control group reported a total mean score ≥20. Among females, the UCLP group showed significantly higher scores in the vertical jaw mobility domain compared to both noncleft groups (*P* = 0.001, 0.005), Table 2.

### Orofacial esthetic scale

A significant difference in mean summary OES scores was observed between the UCLP and Ortho groups (*P* = 0.042), Table 3. A mean summary score ≤ 30 was noted in four subjects in the UCLP group, one in the Ortho group and two in the Control group. Significant differences were noted between the UCLP and Ortho groups in the face, profile, and mouth appearance domains (*P* = 0.012, 0.003, 0.04, respectively). Gender-specific analysis revealed that the difference in profile appearance was present among males, Table 3.

## Discussion

The main findings of this study were that the interdisciplinary treated patients born with UCLP in this cohort perceive their orofacial appearance in a similar way as noncleft peers who have not received orthodontic treatment. Whereas, noncleft orthodontically treated young adults perceive their orofacial appearance more pleasing in the dimensions of the face, profile and mouth appearance. Questionnaires that capture patient’s subjective perception of a condition may offer valuable insight into the patient perspective and enable comparisons across different patient populations. However, it is essential to use questionnaires that are both valid and reliable. In the present study, three validated instruments were used (OHIP-14, JFLS-20, and OES) [20, 21, 23, 24] with the aim to evaluate the self-perceived OHRQoL in treated young adults born with UCLP compared to subjects without a cleft.

The selection of questionnaires in this study was guided by the aim to enable direct comparisons between individuals with UCLP and noncleft peers, both with and without orthodontic treatment. Therefore, we chose to use generic and broadly applicable instruments, OHIP-14, JFLS-20, and OES, which are validated for use across diverse populations. While cleft-specific instruments such as the Cleft-Q offer valuable insight into domains uniquely relevant to individuals with cleft conditions [25], their use is limited to cleft-affected populations and would not have permitted comparisons with our noncleft control groups. Similarly, although the Orthognathic Quality of Life Questionnaires (OQLQ) may be more sensitive to functional and esthetic concerns related to skeletal discrepancies [26], it is not validated for use in individuals without such issues. Future research focusing exclusively on cleft populations may benefit from the inclusion of condition specific tools such as the Cleft-Q, particularly when the objective is to evaluate surgical outcomes or psychological adaptation in greater detail.

Regarding OHRQoL, no significant difference in the OHIP-14 mean summary score was observed between the UCLP group and the two groups born without a cleft, indicating that the cleft, after treatment, does not affect overall OHRQoL, since the total OHIP-14 scores were similar to those of peers born without a cleft. A few subjects in all three groups reported an increased level of oral health-related impact (OHIP-14 score ≥ 15), which might be expected in a general population. However, a significant difference between the groups was noted between the UCLP group and the Ortho group in the dimension of orofacial appearance. When the groups were divided by gender, the difference was limited to the male group.

It should be noted that no specific data were collected regarding whether participants in the UCLP group had undergone or were scheduled to undergo orthognathic surgery. However, no individuals were excluded from the study based on orthognathic treatment status. Therefore, the study cohort is likely to reflect the full range of skeletal presentations typically seen in individuals with UCLP at the age of 19, including those with more severe anteroposterior or vertical maxillary discrepancies. Nevertheless, the absence of subgroup analysis based on orthognathic surgical history represents a limitation, as we cannot assess the potential influence of such treatment on OHRQoL outcomes within the cohort. The mean summary score of the OHIP-14 in the UCLP group was slightly higher (7.3 versus 6.0) than in a Finnish study of 18 year-olds born with either a cleft lip (CL) only or a CLP [27], but somewhat lower (7.3 versus 8.5) than a study of 14.4 year-olds born with CL only or CLP [28]. Overall, it was found that young, treated adults born with UCLP do not have elevated negative impact on OHRQoL compared to noncleft controls. This contrasts with earlier studies reporting poorer OHRQoL in populations with CLP [10, 11, 13], although two of these used different questionnaires, which limits comparability. Both the Finnish study [27] and a Norwegian study [29], which also used the OHIP-14, categorized the impact of oral health differently and did not include noncleft control groups. These differences reduce comparability, although it is notable that the Norwegian study reported a mean summary score of 6.7 for the UCLP group, similar to the findings in our cohort. The authors also reported a significant association between OHRQoL and sagittal intermaxillary discrepancies, an aspect that could not be evaluated in the present study as no clinical data on intermaxillary relations were collected.

The JFLS-20 was used to assess jaw functional limitations. The UCLP group showed a higher mean summery score (8.3) compared to the Ortho (4.8) and Control (6.0) groups, though differences were not statistically significant overall. Some individuals had mean summary scores of ≥20, which were present across all three groups, though more commonly in the UCLP and Control groups.

However, in the domain of vertical jaw mobility, a significant difference was found in the female subgroup, suggesting greater functional limitations among females with UCLP. Although the underlying causes remain speculative, this may reflect sex-based differences in perception, muscle function, or psychosocial sensitivity, and warrants further investigation.

The wide confidence intervals observed for the JFLS-20 mean summary scores in all three groups reflect both the modest sample sizes and the considerable variation in individual responses. This variability may be attributed to differences in functional status or subjective interpretation of jaw function. It underscores the importance of interpreting mean scores with caution, taking into account the underlying distribution of responses. The broad range of reported values highlights the heterogeneity of experiences even within the same group.

Peroz et al. found that adults treated for UCLP reported lower satisfaction with the appearance of their lips, face, and overall appearance compared to a control group without cleft [8]. The study did not consider whether the control group had received orthodontic treatment, which in the present study impacted the outcome and therefore makes it difficult to compare results.

This highlights the importance of considering treatment history when selecting control groups. Unlike previous studies comparing cleft and noncleft populations, we stratified the noncleft comparison groups based on orthodontic treatment status. This distinction revealed that orthodontic treatment in noncleft individuals is associated with improved OHRQoL, related to orofacial appearance, as reflected in higher OES summary scores. Consequently, the finding that young adults with UCLP reported OHRQoL similar to noncleft controls without orthodontic treatment may reflect both the success of interdisciplinary cleft care and the positive influence of orthodontic treatment on perceived orofacial esthetics and overall quality of life. This methodological difference may explain why our findings differ from earlier studies that reported greater negative impact in cleft populations without considering treatment history in controls.

This study has several limitations that should be considered. Due to its cross-sectional design, no causal inferences can be made. We did not collect data on clinical variables such as lateral incisor presence or replacement, or type of space closure, which could influence outcomes. The lack of detailed data regarding these features limits our ability to explore their potential role as confounders. Nevertheless, the study reflects a real-world population treated within interdisciplinary care and likely includes a variety of treatment scenarios. This diversity may enhance generalizability but underlines the need for future studies to stratify based on detailed treatment characteristics.

Additionally, the study was conducted within a single national treatment program in a high-income country, which may limit the generalizability of the findings to other healthcare settings with different treatment protocols or resource availability.

All participants were recruited from the Stockholm public dental care system, including university-affiliated clinics, which may introduce some degree of selection bias.

Orthodontic treatment need in the control groups was determined using clinical indices commonly applied in Stockholm public dental care. The orthodontically treated group included individuals with malocclusions deemed to require treatment based on functional or esthetic indications, while the nontreated group consisted of individuals with no or low registered treatment need. However, it should be noted that different regions in Sweden and in other parts of the world may apply varying indices or thresholds when evaluating treatment indications. This regional variation may influence group allocation and is considered a potential limitation of the study.

The sample size was not calculated for subgroup analyses such as gender comparisons. Therefore, while gender-specific results were included for descriptive purposes, these comparisons may be underpowered, and the findings should be interpreted with caution.

The results of this study may serve as a source of support for families of children born with UCLP. Despite the visible nature of the condition in early life, our findings suggest that, following interdisciplinary treatment, young adults report an OHRQoL comparable to their noncleft peers without orthodontic treatment. This underscores the effectiveness of modern cleft care protocols and may assist clinicians in counseling families, offering a realistic and hopeful perspective on long-term functional and esthetic outcomes.

## Conclusion

Young adults born with UCLP who received long-term interdisciplinary treatment reported overall OHRQoL comparable to that of their noncleft peers without a history of orthodontic treatment. However, noncleft peers who had received orthodontic treatment reported greater satisfaction with their orofacial appearance. These findings highlight the positive long-term outcomes of interdisciplinary cleft care and underscore the importance of considering treatment history when selecting and interpreting control groups. The results may offer support for parents and caregivers, demonstrating that despite early challenges, individuals with UCLP can achieve satisfactory functional and esthetic outcomes by young adulthood.

## Data Availability

The datasets analyzed in the current study are available from the corresponding author on reasonable request

## References

[1] Parham MJ, Simpson AE, Moreno TA et al Updates in cleft care. Semin Plast Surg 2023;37:240–52. 10.1055/s-0043-1776733 38098682 PMC10718659

[2] Paradowska-Stolarz A, Mikulewicz M, Dus-Ilnicka I. Current concepts and challenges in the treatment of cleft lip and palate patients-a comprehensive review. J Pers Med 2022;12:2089. 10.3390/jpm12122089 36556309 PMC9783897

[3] Ayoub A, Bell A, Simmons D et al 3D assessment of lip scarring and residual dysmorphology following surgical repair of cleft lip and palate: a preliminary study. Cleft Palate Craniofac J 2011;48:379–87. 10.1597/10-057 20815731

[4] Peterson P, Mars M, Gowans A et al Mean GOSLON yardstick scores after 3 different treatment protocols-a long-term study of patients with unilateral cleft lip and palate. Cleft Palate Craniofac J 2019;56:236–47. 10.1177/1055665618774010 29738290

[5] Peterson P, Nyberg J, Persson C et al Speech outcome and self-reported communicative ability in young adults born with unilateral cleft lip and palate: comparing long-term results after 2 different surgical methods for palatal repair. Cleft Palate Craniofac J 2022;59:751–64. 10.1177/10556656211025926 34263653

[6] Kallunki J, Sollenius O, Paulsson L et al Oral health-related quality of life among children with excessive overjet or unilateral posterior crossbite with functional shift compared to children with no or mild orthodontic treatment need. Eur J Orthod 2019;41:111–6. 10.1093/ejo/cjy033 29878165

[7] Sabzevari B, Fatemi A, Soleimani M et al Masticatory performance and oral health related to quality of life before and after orthodontic treatment: a systematic review and meta-analysis. Eur J Transl Myol 2024;34:12101. 10.4081/ejtm.2024.12101 38357970 PMC11017174

[8] Peroz R, Hakelius M, Falk-Delgado A et al Patient reported outcome following the skoog unilateral cleft lip repair among adults-a long-term cohort study and comparison to a non-cleft population. Cleft Palate Craniofac J 2024;61:1548–58. 10.1177/10556656231177139 37246371 PMC11323433

[9] Sinko K, Jagsch R, Prechtl V et al Evaluation of esthetic, functional, and quality-of-life outcome in adult cleft lip and palate patients. Cleft Palate Craniofac J 2005;42:355–61. 10.1597/03-142.1 16001915

[10] Sahoo AR, Dheer SS, Mahesh PC et al A questionnaire study to assess patients with cleft lip and palate for their oral health-related quality of life. Cureus 2023;15:e38712. 10.7759/cureus.38712 37292523 PMC10246514

[11] Foo P, Sampson W, Roberts R et al General health-related quality of life and oral health impact among Australians with cleft compared with population norms; age and gender differences. Cleft Palate Craniofac J 2012;49:406–13. 10.1597/10-126 21309686

[12] Marcusson A, Akerlind I, Paulin G. Quality of life in adults with repaired complete cleft lip and palate. Cleft Palate Craniofac J 2001;38:379–85. 10.1597/1545-1569_2001_038_0379_qoliaw_2.0.co_2 11420018

[13] Silva M, Balderrama IF, Wobeto AP et al The impact of nonsyndromic cleft lip with or without cleft palate on oral health-related quality of life. J Appl Oral Sci 2018;26:e20170145. 10.1590/1678-7757-2017-0145 29641750 PMC5912398

[14] Lecommandeur S, de Buys Roessingh A, Dumont L et al Assessment of multiple dimensions of psychological well-being in Swiss youth born with a unilateral cleft lip and palate. Cleft Palate Craniofac J 2025;62:326–36. 10.1177/10556656231219418 38093407 PMC11909769

[15] Tennison CW . The repair of the unilateral cleft lip by the stencil method. Plast Reconstr Surg (1946) 1952;9:115–20. 10.1097/00006534-195202000-00005 14920211

[16] McComb H . Primary correction of unilateral cleft lip nasal deformity: a 10-year review. Plast Reconstr Surg 1985;75:791–9. 10.1097/00006534-198506000-00003 4001197

[17] Mendoza M, Molina F, Azzolini C et al Minimal incision palatopharyngoplasty. A preliminary report. Scand J Plast Reconstr Surg Hand Surg 1994;28:199–205. 10.3109/02844319409015981 7831550

[18] Karsten A, Larson M, Larson O. Dental occlusion after Veau-Wardill-Kilner versus minimal incision technique repair of isolated clefts of the hard and soft palate. Cleft Palate Craniofac J 2003;40:504–10. 10.1597/1545-1569_2003_040_0504_doavvm_2.0.co_2 12943437

[19] Boyne PJ, Sands NR. Secondary bone grafting of residual alveolar and palatal clefts. J Oral Surg 1972;30:87–92. https://pubmed.ncbi.nlm.nih.gov/4550446/4550446

[20] Larsson P, John MT, Hakeberg M et al General population norms of the Swedish short forms of oral health impact profile. J Oral Rehabil 2014;41:275–81. 10.1111/joor.12137 24447237

[21] Ohrbach R, Larsson P, List T. The jaw functional limitation scale: development, reliability, and validity of 8-item and 20-item versions. J Orofac Pain 2008;22:219–30. https://pubmed.ncbi.nlm.nih.gov/18780535/18780535

[22] Larsson P, John MT, Nilner K, et al Reliability and validity of the Orofacial Esthetic Scale in prosthodontic patients. Int J Prosthodont 2010;23:257–62. https://pubmed.ncbi.nlm.nih.gov/20552093/20552093

[23] Larsson P, John MT, Nilner K, et al Development of an Orofacial Esthetic Scale in prosthodontic patients. Int J Prosthodont 2010;23:249–56. https://pubmed.ncbi.nlm.nih.gov/20552092/20552092

[24] John MT, Larsson P, Nilner K et al Validation of the Orofacial Esthetic Scale in the general population. Health Qual Life Outcomes 2012;10:135. 10.1186/1477-7525-10-135 23158767 PMC3534548

[25] Klassen AF, Riff KWW, Longmire NM et al Psychometric findings and normative values for the CLEFT-Q based on 2434 children and young adult patients with cleft lip and/or palate from 12 countries. CMAJ 2018;190:E455–62. 10.1503/cmaj.170289 29661814 PMC5903887

[26] Ahl M, Marcusson A, Ulander M et al Translation and validation of the English-language instrument orthognathic quality of life questionair into Swedish. Acta Odontol Scand 2021;79:19–24. 10.1080/00016357.2020.1768284 32432962

[27] Corcoran M, Karki S, Harila V et al Oral health-related quality of life among young adults with cleft in northern Finland. Clin Exp Dent Res 2020;6:305–10. 10.1002/cre2.284 32396275 PMC7301391

[28] Antoun JS, Fowler PV, Jack HC et al Oral health-related quality of life changes in standard, cleft, and surgery patients after orthodontic treatment. Am J Orthod Dentofacial Orthop 2015;148:568–75. 10.1016/j.ajodo.2015.03.028 26432312

[29] Saele PK, Mustafa M, Astrom AN. Orthodontic status and association with oral-health-related quality of life-a study of 16-year-old Norwegians with a cleft lip and palate. Int J Environ Res Public Health 2024;21:550. 10.3390/ijerph21050550 38791765 PMC11121370

